# Insanity among the Convicts at Pentonville, Milbank, and Dartmoor Prisons

**Published:** 1857-04-01

**Authors:** 


### INSANITY AMONG THE CONVICTS AT PENTONVILLE, MILBANK, AND DARTMOOR PRISONS.

We extract the following interesting particulars from the last Parliamentary Report of the *Directors of Convict Prisons.* With respect to Pentonville, Mr. C. L. Bradley, the highly intelligent and active medical officer, reports as follows:—

Two cases of insanity have been removed to Bethlehem Hospital; the following are the particulars thereof:—H. G., 5960, attracted notice on admission by a peculiarity of manner, and in a short time afterwards his conduct and conversation were sufficiently strange to lead to doubts being entertained of his sanity. He was placed under observation and treatment in the infirmary, and eventually it became apparent that he was the subject of insane delusions. The result of inquiries into the previous history of the prisoner were at first obscure and contradictory; at length the following reliable account was obtained from the clergyman of his parish:—the prisoner, he stated, had been formerly an industrious and respectable man, a regular attendant at his church, but after sustaining heavy pecuniary losses, became dejected and "perplexed himself by an over-curious reading of the Bible," and finally, without any assignable motive, fired a neighbour's haystack. Connecting this history with the delusions exhibited by the prisoner in Pentonville, there is much reason to believe that the crime of arson, with which he was charged, was committed under the influence of mental disease, but which required long-continued and close observation for its detection.

G. W., 6044, was received into Pentonville to undergo a second probationary period of separate confinement for extreme idleness and insubordination at the public works. From the first he refused to work, and obstinately persisted in that determination. He was at times violent and generally abusive; occasionally his conversation was such as to lead one to hesitate whether to refer it to audacity and impudence, or to actual incoherence. The character given him by those who had had charge of him in other prisons was to the effect that he was idle, ill-conducted, and an impostor. He was placed in the infirmary under close surveillance for some months without any marked change, except that his conversation became more incoherent; but there were still many features, both in the history and general aspect of the case, which for a time justified a grave suspicion that the incoherence was simulated. At length, however, more decided indications of mental disease were developed, and the prisoner was removed from the prison as a lunatic. In the above cases, two of the most difficult to pronounce an opinion upon of any I recollect to have occurred during a medical experience of thirteen years at this prison, I had the advantage of obtaining the able assistance of Dr. Forbes Winslow, whose name as a psychologist is so well known.

F. M., 5695, a prisoner sent here to undergo a second period of separate confinement, exhibited soon after his admission an irritable state of mind, and entertained groundless suspicions as regards treatment by the officers. At one time he maintained that suffocating vapours were introduced at night into his cell through the air-flues. Under the treatment employed the delusion disappeared, and his mind was restored to a healthy condition.

Four cases of "mental irritability" are noticed in the tables (p. 36). In two cases the prisoners suffered from dyspepsia, and imagined that their illness was caused by deleterious substances clandestinely mixed by the officers with the food supplied to them. The third fancied he was in the possession of an important secret connected with religious subjects, which he refused to disclose. The fourth asserted that he was made unhappy by being suspected and pointed out by the officers as guilty of vicious practices. Although it would appear from the above statement that delusions were observed in these cases, yet they were not sufficiently "fixed" or absurd to be regarded as *insane* delusions, but, nevertheless, were indications of a mental condition, which, under adverse circumstances, would probably have terminated in actual mental disease. By suspending the discipline of separation, and using suitable moral and medical treatment, recovery to health was obtained, and the mind regained its equilibrium.

W. G., 5971, hanged himself in his cell in the fourteenth month of his imprisonment here. He had never exhibited any indication of mental affection, and his health during the whole period of his confinement was perfect. He was industrious at his trade, and had made considerable progress under the schoolmaster. He had never been punished for a prison offence. After his death, it was discovered that he had indulged the hope of soon regaining his liberty, which was annihilated by the notices recently issued to prisoners sentenced to penal servitude. The unexpected prospect of a protracted imprisonment may with great probability be regarded as having in the above instance led to the commission of an act of suicide.

Two Chinamen attempted to hang themselves in the cells shortly after their admission, but were saved from harm by the timely interference of an officer. They were removed from the prison as unfit subjects for separate confinement.

Two prisoners attempted suicide by strangulation while under punishment in the dark cells. The prisoners stated that they were tried of life, and one was ascertained to have attempted suicide in other prisons twice before.

There were also three cases in which the attendant circumstances rendered it morally certain that the attempts were feigned, with a view to excite sympathy or create alarm.

Whenever the medical officer had reason to believe that "separation" was likely to act injuriously upon the mental or bodily health, the discipline was suspended, and the prisoner permitted to work in association with others. The total number requiring the medical officer's interposition in this respect was 23; 10 on account of ill health, and 13 on mental grounds. The above measure in some cases no doubt averted positive injury, but was in all cases attended with benefit.

Three prisoners were removed from the prison on mental grounds shortly after admission—namely, the two Chinamen before noticed as having attempted suicide, and P. M., an excitable Irishman, who had formerly been a hard drinker, and had severely injured his skull.

Of Milbank, Dr. Baly reports:—The account of mental diseases amongst the prisoners during the past year is a favourable one. Six men were removed to Bethlehem; and two other men who were insane for a short period, recovered in the prison. Three of those removed were insane in 1854 (one of them received insane), and they were included in last year's return. Of the other three, M. I. was insane when he was received; another, T. H., was believed to be an imposter, but, on account of his extraordinary conduct, it was deemed right to submit his case to the judgment of the physician practising in the department of lunacy, who gave a certificate of his insanity, and he was removed to Bethlehem Hospital; but he exhibited no symptom of insanity in that establishment, and has been returned to the prison. The third, J. S., was suddenly attacked with mania three months after his reception, without any apparent cause.

The two men who became insane in the prison but recovered under treatment there, were both of very weak intellect when they were received. One of them, J. H., while recovering from cholera, was seized with epilepsy, from which he had suffered previously, and was left by the epileptic attack in a state of dementia, which continued about seven weeks. The other, F. B., was attacked with epilepsy, according to his statement for the first time in his life, on the day of his reception from Perth, where he had been ten months in separate confinement, and five days afterwards he was seized with acute mania; he recovered in little more than a fortnight. The cases of J. S. and F. B. are therefore the only ones occurring during the past year that can with any probability be referred to the influence of imprisonment.

Dr. J. Campbell, the medical officer of the Dartmoor Prison, reports as follows, respecting the mental condition of those under his kind care:—

Chronic diarrhœa has been the cause of removal to this place in several instances, and as the complaint has commonly been of long standing or complicated with disease of the lungs, it has proved very intractable. Two of the invalids received during the year for this disease have died of pulmonary consumption, and a third is now in a precarious state from the disease itself.

*Scrofula.*—Considering the great number of prisoners labouring under scrofulous diseases, the cases admitted into the infirmary have not been numerous. The most common form has been suppurating glands of the neck, but there were also several cases of diseased bones and joints, also large abscesses about the chest and lower extremities. Some of these cases proved tedious from impairment of the constitution; but when unattended by any marked disease of the internal organs, the change to this place soon exerted a beneficial influence; and most of the cases are either progressing favourably or have terminated in a satisfactory manner.

From there being upwards of 80 prisoners in this establishment of weak or doubtful mind, such cases are frequently brought to my notice. Although few appear on the sick list for mental affections, we have commonly about half-a-dozen in the infirmary for the treatment of other diseases, particularly catarrh, diarrhœa, and epilepsy, those being the complaints to which this class of invalids appear peculiarly liable. When suffering from bodily disease, those men are generally more troublesome, and subject to fits of excitement which render the greatest vigilance necessary. Two cases have been recommended for removal during the year:—1st, No. 2,665, R. D., was received from Portland Prison, September 6th, 1854, for "imbecility of mind, violent, and intractable." He maintained the same character here, and as he was several times in the hospital for the treatment of slight bodily ailments, as well as for observation, I had frequent opportunities of watching him, which confirmed my opinion of his insanity. As he was guilty of repeated acts of violence and assaults upon officers, the last being an attempt to knock an officer down with a spade, I deemed it necessary to recommend his removal to a lunatic asylum. 2nd, No. 3,143, J. J., was received from Northampton Gaol in August, 1855, the cause of removal being "weak mind, and unfit for separation;" and his whole conduct was so eccentric and violent as to leave no doubt in my mind of his insanity. His manner was very peculiar, and he was guilty of various serious and dangerous acts; such as burning his Bible, prayer, and other books in his cell, stating as his reason, "he could not derive any benefit from them;"

breaking articles of furniture in the cell, as well as the window, without the slightest provocation, and frequently threatening the lives of the officers. It was considered unsafe to allow him to associate with other prisoners, and an inquiry into the state of his mind resulted in an order for his removal to Bethlehem.

A great many of the weak-minded prisoners are, however, harmless, and even industrious, which reflects great credit on the careful management of the officers; but, as I have before observed, there appears to me a necessity for a more complete separation between the weak-minded and healthy prisoners, as their eccentricities give rise to remarks which irritate and excite them. It would also bring them more prominently under the notice of the officers in charge of the working parties.

Mental affections have been rather on the increase during the year, the number of admissions being forty, which, added to those remaining at the close of 1854, give a total of 105. Such cases must, at all times, be a source of anxiety to those in charge, as even when the malady is in its mildest form, such as eccentricity or incoherency, they are liable to become excited and violent when least expected, and necessarily require the most careful supervision.

